# Two Anatomically and Computationally Distinct Learning Signals Predict Changes to Stimulus-Outcome Associations in Hippocampus

**DOI:** 10.1016/j.neuron.2016.02.014

**Published:** 2016-03-16

**Authors:** Erie D. Boorman, Vani G. Rajendran, Jill X. O’Reilly, Tim E. Behrens

**Affiliations:** 1 Centre for Functional Magnetic Resonance Imaging of the Brain, University of Oxford, John Radcliffe Hospital, Oxford OX3 9DU, UK; 2 Wellcome Trust Centre for Neuroimaging, Institute of Neurology, University College London, 12 Queen Square, London WC1N 3BG, UK; 3 Max Planck UCL Centre for Computational Psychiatry and Ageing Research, Russell Square House, 10-12 Russell Square London WC1B 5EH, UK

## Abstract

Complex cognitive processes require sophisticated local processing but also interactions between distant brain regions. It is therefore critical to be able to study distant interactions between local computations and the neural representations they act on. Here we report two anatomically and computationally distinct learning signals in lateral orbitofrontal cortex (lOFC) and the dopaminergic ventral midbrain (VM) that predict trial-by-trial changes to a basic internal model in hippocampus. To measure local computations during learning and their interaction with neural representations, we coupled computational fMRI with trial-by-trial fMRI suppression. We find that suppression in a medial temporal lobe network changes trial-by-trial in proportion to stimulus-outcome associations. During interleaved choice trials, we identify learning signals that relate to outcome type in lOFC and to reward value in VM. These intervening choice feedback signals predicted the subsequent change to hippocampal suppression, suggesting a convergence of signals that update the flexible representation of stimulus-outcome associations.

## Highlights

• Probe and choice trials assess the encoding and updating of an internal model
• Learning signals are found in orbitofrontal cortex (OFC) and ventral midbrain (VM)
• Probe trials reveal the model’s neural instantiation in the medial temporal lobe
• Dynamic changes to this instantiation are predicted by OFC and VM learning signals

## Teaser

Boorman et al. reveal neural encoding of an internal model comprising probabilistic transitions between visual stimuli and reward types in a medial temporal lobe network. Changes to its encoding are predicted by two distant computationally and anatomically distinct learning signals.

## Introduction

Behavioral neuroscience has made recent dramatic strides through the integration of formal models of behavior with the measurement of neural signals (Daw et al., 2011, Iglesias et al., 2013, Takahashi et al., 2011). By designing situations in which key learning variables change through the experiment and discovering signals in the brain that fluctuate in the same manner, scientists have been able to draw deep inferences about the types of computations that must underlie behavioral control in different situations. In studies of learning, for example, scientists have been able to dissect intermediary processes into distinct computations, such as prediction errors, volatility or uncertainty estimates, and volatility prediction errors, across several domains of cognition (Behrens et al., 2007, Behrens et al., 2009, Boorman et al., 2013a, Cooper et al., 2010, D’Ardenne et al., 2008, Daw et al., 2011, den Ouden et al., 2009, Hampton et al., 2006, Hare et al., 2008, Iglesias et al., 2013, Klein-Flügge et al., 2011, Payzan-LeNestour and Bossaerts, 2011). The identification of behavioral and neural correlates of such learning signals has been influential because they imply particular intermediary computations that are performed in the course of learning.

However, despite the computational insight bestowed from identifying one type of learning signal or another, outside of striatal dopamine (Collins and Frank, 2014, Jocham et al., 2011, Kravitz et al., 2012), little is known about how these different signals are used in the brain. When a prediction error is signaled, for example, how does it change the brain’s representations of the task variables that will determine future behavior? In short, how do these learning signals cause learning? A major hurdle to answering such questions is that the brain regions that generate learning signals are not necessarily the same regions where the learning occurs. Signals broadcast from projection neurons in one region may have effects on neural representations in another.

In order to study this type of question, it is therefore imperative to develop techniques that act simultaneously at different scales—capable of recording data across multiple brain regions simultaneously, but also capable of indexing neural representations within a brain region and how they change with learning. In human neuroscience, despite ambiguity concerning the underlying biophysical mechanism, repetition suppression (RS) fMRI is a well-validated technique that, when combined with careful experimental design, allows inferences to be made about the underlying neuronal representations. A recently developed variant to RS, cross-stimulus suppression (CSS), has been used to show that blood-oxygen-level-dependent (BOLD) suppression can be measured not to repetition of a stimulus feature or percept, but instead to pairs of stimuli related through association, when the stimuli have been deterministically paired and well learned (Klein-Flügge et al., 2013, Meyer and Olson, 2011). If such techniques were combined with the computational approaches discussed above, it should be possible not only to measure both the learning signals and the task representations but also the impact of different learning signals on task representations.

Here, we develop a task that requires subjects to keep track of stochastic transitions between particular stimuli and outcome identities—a basic internal model—in order to maximize reward. The task induces two learning signals simultaneously, one for learning from reward value and one for learning reward-size-independent stimulus-outcome associations that respectively relate to neural signals in the dopaminergic ventral midbrain (VM) and lateral orbital frontal cortex (lOFC). We interleave this task on a trial-by-trial basis with blocks of CSS fMRI to measure the current neural representation of the internal model and find its instantiation in the hippocampus, amygdala, and surrounding and interconnected cortex. Critically, this instantiation changes on a trial-by-trial basis, and this change is predicted by the intervening lOFC signal at the learning event. Furthermore, the VM signal also predicts this change, but only in subjects who will be (inaccurately) influenced by reward in their behavior. This implies that associations that are critical for building internal models of the world can be stored in the medial temporal lobe system and reflect computational changes during learning that are signaled from distant structures.

## Results

### Task

**Figure 1:**
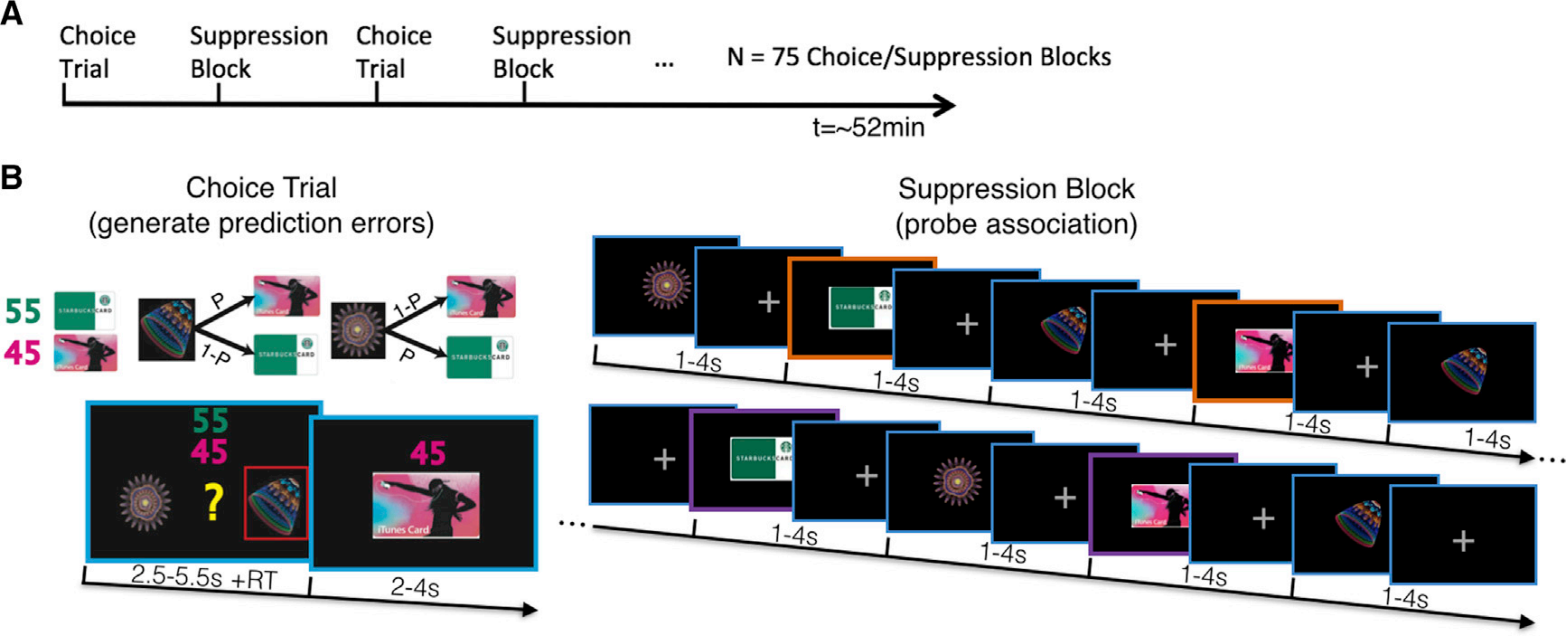
Experimental Timeline and Task (A) Experimental timeline. Single choice trials and suppression blocks were interleaved 75 times during the experiment. (B) Left: Choice trial. Potential reward payouts were paired deterministically with either of two gift cards, as indicated by the two numbers’ colors. Subjects were instructed to select between two abstract stimuli presented on the basis of two pieces of information: the estimated likelihood that a stimulus would lead to either of the gift card outcomes if selected, which could be learned from choice feedback, and the amount of points (sampled from a uniform distribution with a range of 1–100) that could be won on each gift card, which changed randomly from trial to trial. Right: Suppression block: stimuli and outcomes were presented in an interleaved, pseudorandom order, totaling nine items per block (one example block is shown). During suppression blocks, subjects were unpredictably probed and asked to report which item they had seen last. By deducting £1 from their total earnings for incorrect responses, we incentivized participants to attend to each item presented. Those outcomes that were preceded by a high-contingency stimulus, based on learning during the choice trials up until that suppression block, are highlighted by an orange frame, while those preceded by a low-contingency stimulus are highlighted by a purple frame.

We hypothesized that we could measure neural updates to stimulus-outcome identity associations and probe those recently updated associations by interleaving CSS blocks with single choice trials. During choice trials (Figure 1), randomly generated potential reward payouts were paired with either of two gift cards. These potential payouts were manipulated independently from the likelihood that each of two shape stimuli would lead to either of two gift cards, if chosen. This manipulation meant that it was advantageous to learn the transition probabilities from shape stimuli to gift card outcomes but not about the reward amount obtained on a gift card, since these changed randomly from trial-to-trial. The task structure encouraged participants to first select the more desired gift card goal based on the current potential payouts and then reverse-infer the stimulus they believed would most likely lead to that desired outcome.

To probe the neural encoding of specific associations as they were acquired and updated through learning in choice trials, but in the absence of potential confounds during choice and feedback events, choice trials were interleaved with CSS blocks. During CSS blocks, participants observed individual presentations of either a stimulus or a gift card, in alternating order, and were incentivized to attend to each item presented (Figure 1). In each CSS block, each stimulus-outcome transition was presented once, in pseudorandom order, totaling nine single-item presentations. This feature of the design enabled us to compare gift card presentations preceded by high-contingency (HC) stimuli (based on learning during choice trials up to the current CSS block) with those preceded by low-contingency (LC) stimuli. This procedure also ensured any incidental learning during CSS blocks should equate on average, since each possible transition was presented with equal frequency during a CSS block.

### Behavior

**Figure 2:**
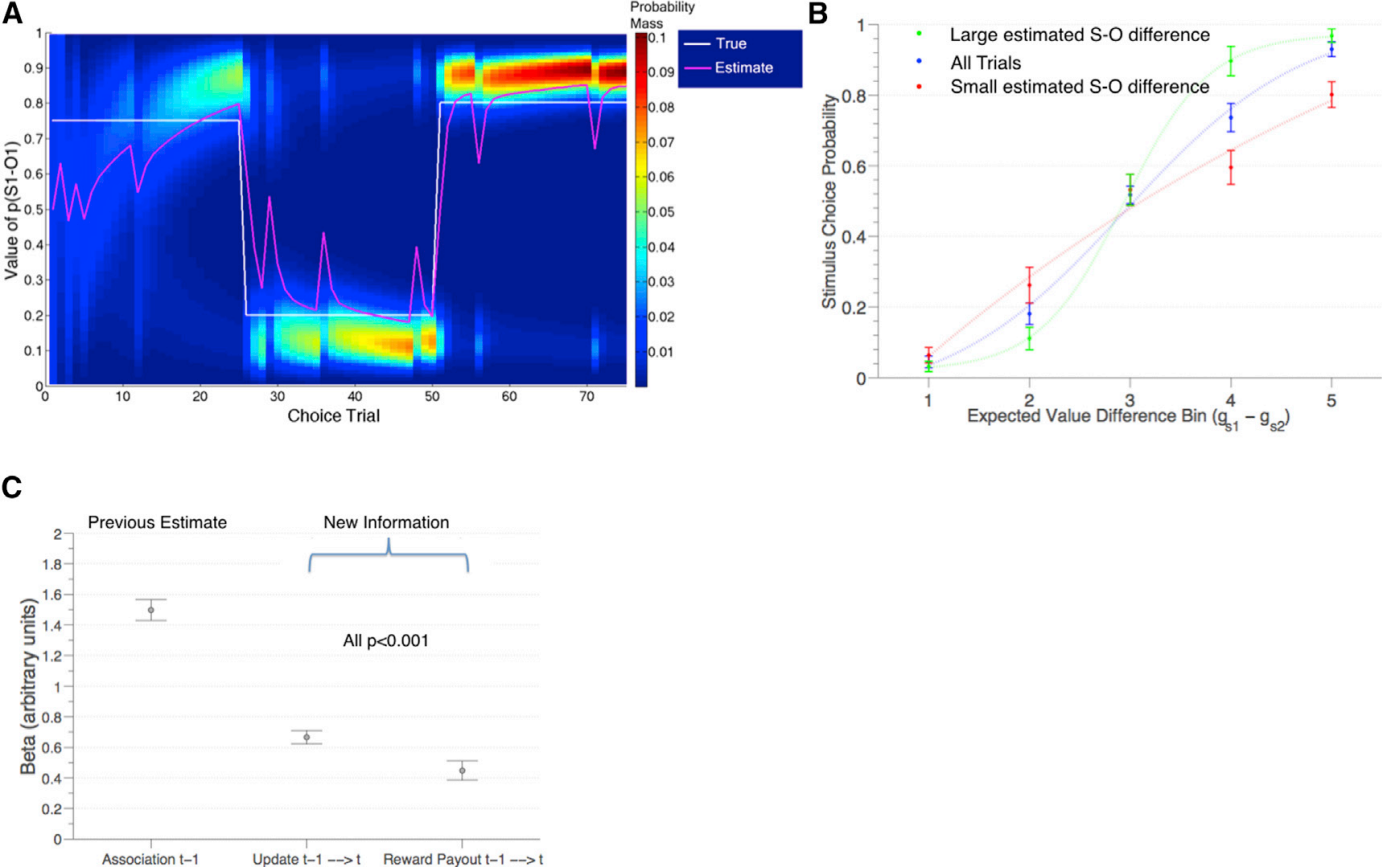
Model Parameters and Behavior (A) Heatmap depicts probability mass of each value of the distribution over transition probabilities between a stimulus 1 and outcome 1 on each choice trial. The true transition probability is shown in white. The mean of the distribution is shown in magenta, which forms our estimate of participants’ current belief in the association strength. The true probabilities change twice during the experiment. (B) Frequency of selecting stimulus1 (arbitrarily defined) is plotted against five equally distributed value difference (*g*_*s1*_ − *g*_*s2*_) bins for all trials (blue), trials for which the model’s estimate in S-O transition probabilities $\left(\text{abs}\left(r_{S1\to O1}-r_{S2\to O1}\right)\right)$ is larger (green; >60^th^ percentile; i.e., when the magenta line in Figure 2A is either high or low) and smaller (plotted in red; <40^th^ percentile; i.e., when the magenta line in Figure 2A is close to 0.5). Sigmoidal functions are plotted through the means of the five bins. The slope of the sigmoidal function is steeper when the difference in estimated transition probabilities is larger but shallower when the difference is smaller. Circles denote group mean and error bars ± SEM. (C) Mean ± SEM of regression coefficients resulting from multiple regression analysis of stimulus1 choices based on three explanatory variables defined with respect to the subject’s more desired outcome on the current trial; left: the previous estimate of the association of the stimulus with that desired outcome (computed from *r*, illustrated in magenta in [A]); middle: the update to that association (computed from the latest feedback); right: the reward payout obtained at the latest feedback on the association between stimulus 1 and the currently desired outcome. All *t*(21)>5.0, p < 0.001, one-sample t test. See also Figures S1–S3.

To generate trial-by-trial predictions of participants’ beliefs about the stimulus-outcome transition probabilities, and updates to those beliefs, to regress against behavior and BOLD responses, we constructed a normative Bayesian reversal-learning model (Figure 2A; see Supplemental Information and Figure S1 for a detailed description and illustration of joint distributions). The purpose of the model was to generate trial-by-trial predictions to relate to neural responses during CSS blocks and choice feedback, rather than to optimally capture behavior. Nonetheless, this model outperformed several alternative models, including an established, previously described Bayesian volatility model that has been shown to capture behavior well in tasks with similar structure (Behrens et al., 2007) (Table 1).

We first examined the relationship between stimulus choices and their expected values ($g_{s1}$, Equation 9), as estimated by the best-fitting Bayesian reversal learning model (Figure 2B). We observed a relatively steep sigmoidal relationship, suggesting that on average the model accurately captured fluctuations in subject choices. This relationship was confirmed by logistic regression analysis of subject choices, using model estimates of expected value as predictors, without any free parameters fit to behavior: *t*(21) = 9.48, p < 0.0001 (one-sample t test). To further examine the relationship between subject choices and model estimates of transition probabilities, we have (i) plotted sigmoidal choice functions when the difference between transition probabilities was high (> 60^th^ percentile) or low (< 40^th^ percentile), which revealed an expected reduction in the sigmoidal function’s slope (Figure 2B), and (ii) plotted choices over the course of the experiment alongside transition probability estimates (Figure S2).

To test which variables at choice feedback drove learning, we performed an analysis designed to isolate the information contained in individual choice outcome events. In addition to the previous association strength and new stimulus-outcome update, our paradigm enabled us to test whether especially large or small reward might additionally influence future choices, though suboptimal in the context of the task. Multiple linear regression revealed that the previous estimate of the stimulus-outcome association strength, the most recent Bayesian update to that association, and the most recent monetary payout, defined as the amount of points obtained on the latest choice outcome, all had a strong and significant positive influence on current stimulus choices (one-sample *t test*: all *t*(21)>5.0, p < 0.001; Figure 2C; Supplemental Information, GLM1). Positive effects of the first two terms show that (i) the more strongly a stimulus was previously associated with a participant’s more preferred outcome on the current choice trial and (ii) the larger the Bayesian update to that association from the latest feedback, the greater the likelihood of selecting that stimulus on the current choice trial. The positive effect of reward payout further indicates that especially large reward effectively “stamped in” updates to stimulus-outcome transitions following a confirmatory outcome, while especially small reward produced even greater changes to beliefs about stimulus-outcome associations following a disconfirmatory outcome.

On each choice trial, one stimulus-outcome association was directly observed, and the other inferred based on the subject’s knowledge of the inverse relationship between stimuli and outcomes dictated by the task structure. To test whether observed and inferred outcomes were differentially weighted during learning, we constructed a variant of the Bayesian reversal-learning model with an additional free parameter that captured the relative weighting of experienced and inferred choice outcomes (Supplemental Information). Values for this fitted weighting parameter did not provide evidence for differential learning from observed or inferred outcomes (mean η = 1.01, one-sample t test against the null hypothesis of no difference [i.e., η = 1]: *t*(21) < 1, p > 0.2), suggesting participants weighed directly observed and inferred updates similarly.

### CSS Reveals Neural Representation of Trial-by-Trial Stimulus-Outcome Association Strength

**Figure 3:**
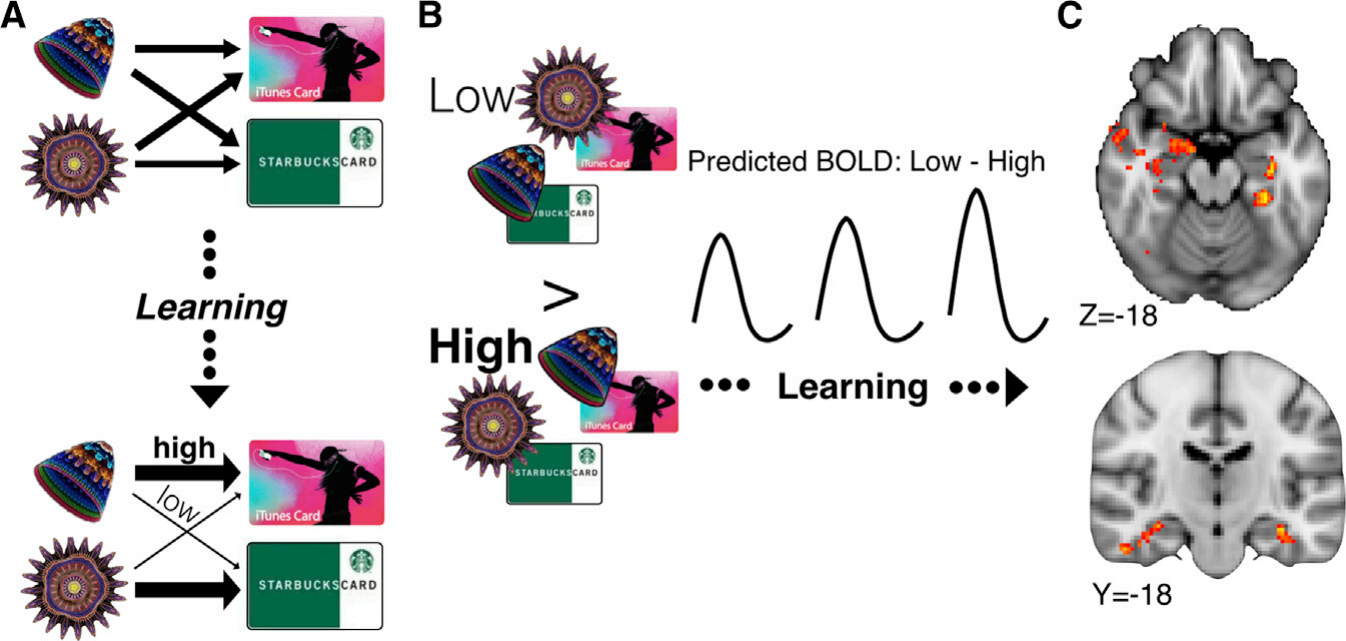
fMRI Results during Cross-Stimulus Suppression Blocks (A) In each CSS block, participants learned that one transition probability from stimulus to gift card was more likely (high) and the other less likely (low) from trial-and-error feedback. We defined high-contingency and low-contingency transitions between stimuli and outcomes based on the computational model’s current estimate of participants’ beliefs in the association strength from choice trials (*r*(t)). (B) We predicted a suppressed BOLD response when the same outcome presentation was preceded by a high-contingency stimulus, compared to a low-contingency stimulus. Further, we computed the difference between BOLD suppression on low and high contingency outcome presentations and regressed this difference against the model-predicted difference in association to produce maps shown in (C). We predicted an increased difference between BOLD responses on low-contingency and high-contingency presentations as the association strength grew (black traces). (C) Axial and coronal slices through Z-statistic maps relating to the effect of the current stimulus-outcome identity association at the time of item presentation during suppression blocks in bilateral hippocampus, parahippocampal gyrus, perirhinal cortex, inferior/middle temporal gyrus, and right amygdala. Activations survived a cluster-forming threshold across the whole brain of Z > 2.3 and a family-wise error rate of p = 0.05.

To probe the flexible encoding of stimulus-outcome identity associations, before and after updating during choice trials, we interleaved CSS blocks and choice trials. In particular, after each choice trial, we compared presentation of gift cards that followed stimuli with which they were more strongly associated (high contingency [HC]) to those that followed stimuli with which they were less strongly associated (low contingency [LC]), based on the associations acquired during choice trials up until the presented CSS block (Figure 3A; see Experimental Procedures). This procedure meant that the BOLD responses evoked by identical gift cards during CSS blocks were compared, differing only in the strength of association with the preceding stimulus presented. Each possible pairing of a stimulus and gift card was presented twice in each block (see Figure 1B), thereby minimizing any potential incidental learning of stimulus-outcome associations during these blocks, since in each block each possible S-O transition was experienced with equal frequency and any incidental learning should be equated between different pairs on average. Based on previous demonstrations of increased suppression for associated, compared to non-associated stimulus-reward or stimulus-stimulus pairs, albeit in the absence of any online learning (Klein-Flügge et al., 2013, Meyer and Olson, 2011), we predicted a reduction in the BOLD response for HC items when compared to LC items. We made the further quantitative prediction that the difference in the degree of CSS between LC and HC items should be proportional to the difference in association strength between LC and HC stimulus-gift card pairs, acquired and updated through learning during choice trials (Figure 3B). To test this prediction, we regressed the current association strength, estimated by the normative Bayesian reversal-learning model, against the difference in BOLD suppression between LC and HC items. This whole-brain analysis identified distributed effects with peaks in bilateral hippocampus and parahippocamal gyrus, right perirhinal cortex, inferior/middle temporal gyrus, and right amygdala, and additional clusters in posterior cingulate cortex and left temporo-parietal junction area (Z > 2.3, p = 0.05 cluster-corrected; Figure 3C; Table S2). The degree of suppression in between choices in these regions therefore flexibly tracked the current on-line association between particular stimuli and outcomes, suggesting a substrate for the online neural representation of a basic internal model composed of transitions between particular visual stimuli and reward outcomes.

To explore whether this network depended on whether the transition observed during CSS blocks was directly experienced or inferred in the previous choice trial, we constructed a separate GLM with these two category of CSS item presentations separately modeled. Contrasting experienced and inferred transitions did not reveal any significant differences, consistent with the absence of any behavioral differences. This null result should be interpreted with caution because of the large asymmetry between the frequencies of directly experienced HC and LC transitions, with far fewer of the latter by design and therefore low efficiency to test this contrast.

### Neural Signatures of Updating during Choice Feedback Events

**Figure 4:**
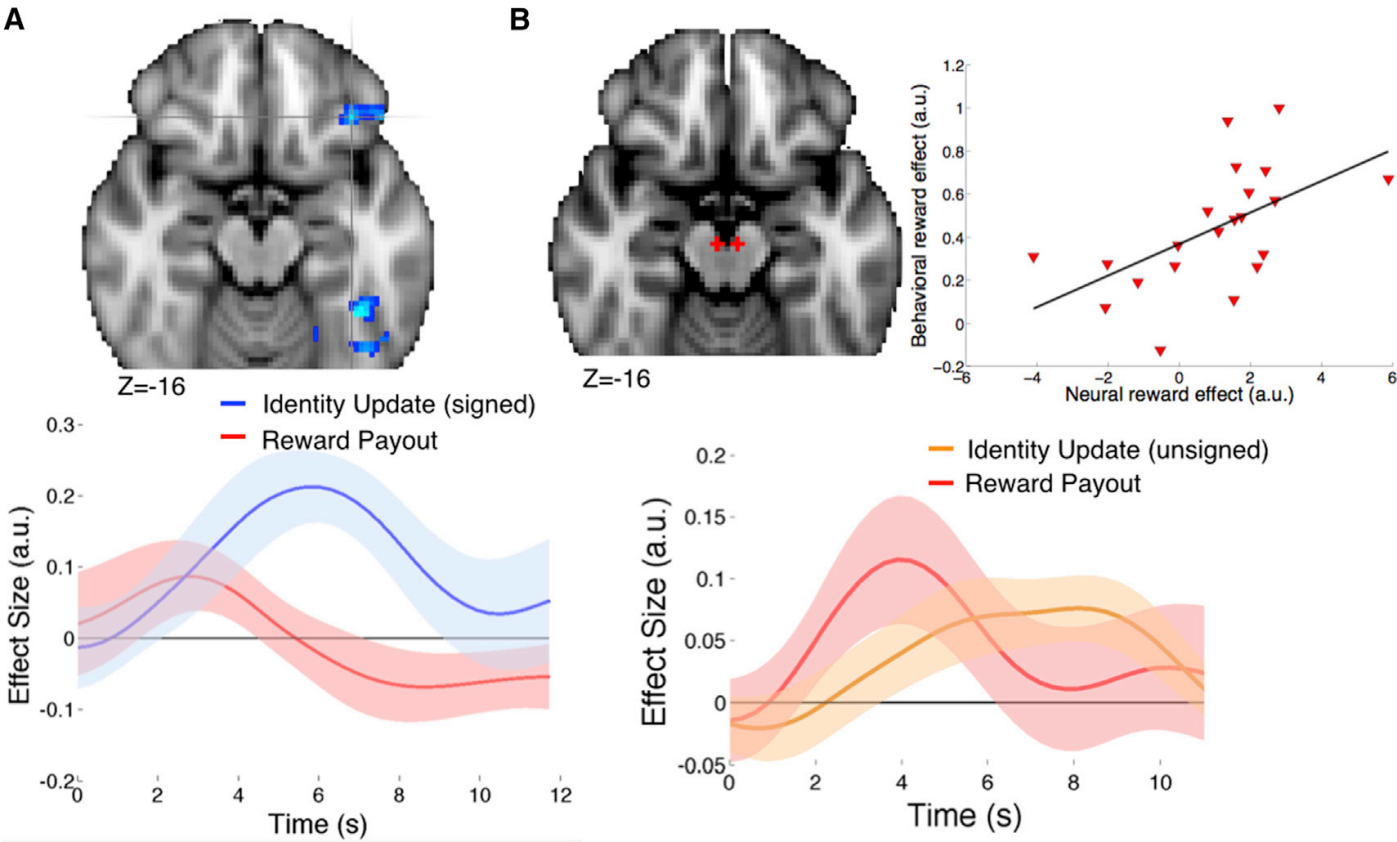
Learning Signals during Choice Trials (A) Top: Axial slice through Z-statistic map displaying effect of signed stimulus-outcome (signed *D*_*KL*_) at feedback of choice trials. Maps display lOFC (crosshairs) and inferior temporal gyral clusters that survived a cluster-forming threshold across the whole brain of Z > 2.3, and a family-wise error rate of p = 0.05. Bottom: Time course of stimulus-outcome identity update and reward payout in independently defined left lOFC region, plotted from feedback onset (for display purposes only). (B) Upper left: ROIs in VM defined from coordinates in Klein-Flügge et al. (2011). Bottom: Time course of unsigned stimulus-outcome identity update (*t*(21) = 2.39, p = 0.01, one-sample t test) and reward payout size (*t*(21) = 2.01, p < 0.05; one-sample t test) in left VM ROI. Upper right: Scatterplot depicts relationship across participants of behavioral and neural effects of reward size in left VM (partial Pearson’s correlation controlling for behavioral stimulus-outcome update effect and previous association before update: ρ = 0.63, p < 0.005). See also Figures S3–S5.

Having identified a network that encoded the online associations during probe CSS trials, we sought to identify learning-related updating signals at feedback during choice trials and to test whether these would explain changes to the network. Our behavioral analysis indicated that both the stimulus-outcome update and the recent reward size impacted learning, motivating tests to identify neural correlates of trial-by-trial fluctuations in these terms at the time participants witnessed choice feedback—the critical time for learning to take place. Notably, interference and recording evidence across species suggests a key role for lOFC in learning and/or using stimulus-outcome identity associations to guide choice (Buckley et al., 2009, Gremel and Costa, 2013, Jones et al., 2012, McDannald et al., 2011, Noonan et al., 2012, Rudebeck and Murray, 2014, Rudebeck et al., 2013b, Rushworth et al., 2011, Stalnaker et al., 2014, Takahashi et al., 2011, Walton et al., 2010, Wilson et al., 2014), supporting the hypothesis that lOFC may be important for updating beliefs about likely reward outcomes. We defined the stimulus-outcome belief update as the Kullback-Liebler divergence (*D*_*KL*_) between posterior and prior beliefs, computed over the distribution of possible transition probabilities, having witnessed a new choice-outcome transition. Here, the *D*_*KL*_ encodes the information contained in the belief update, and has also been termed “Bayesian surprise” (Itti and Baldi, 2009) (see Equation 13 in Supplemental Information and Figure S1). To identify regions whose activity reflected both the size of the stimulus-outcome update and its direction, we signed the *D*_*KL*_ based on each subject’s estimated goal on each trial (where the goal was defined by estimating subject-specific indifference points between gift cards; see Equation 12 in Supplemental Information), such that positive updates corresponded to shifting beliefs toward a subject’s current goal and negative updates corresponded to shifting beliefs away from their goal (Experimental Procedures GLM3). Consistent with our a priori hypothesis, this whole-brain analysis revealed stimulus-outcome update effects in lateral OFC/ventrolateral prefrontal cortex (VLPFC) and also a distributed network including anterior cingulate cortex, inferior temporal cortex, and posterior cingulate cortex (Z > 2.3, p = 0.05 cluster-corrected; Figures 4A and S3; Table S2). Activity in these regions thus reflects how much to update beliefs about the transition probabilities that map stimulus choices to potential outcomes and in which direction, toward or away from a subject’s goal. Notably, this activity cannot be explained by a reward prediction error, because unlike the effect in VM described below, it is unaffected by the magnitude of the reward (mean group effect: *t*(21)<2, p > 0.1; partial correlation between behavioral and neural reward effects, controlling for the behavioral effects of the previous S-O association and the S-O update: ρ = 0.33, p > 0.10; Figure 4A). Rather, it is a learning signal about the identity of the outcome but is signed according to the subject’s current goal or the current focus of the subject’s attention. It is also important to note that these effects cannot simply be explained by increased BOLD responses to confirmatory relative to disconfirmatory outcomes, which were modeled separately in the general linear model (GLM) (see Experimental Procedures, GLM3; Figure S3D). Conversely, the unsigned *D*_*KL*_ term, corresponding to the magnitude of the belief update, independent of its direction, instead recruited a dorsal frontoparietal network, consistent with previous findings related to unsigned state prediction errors during latent learning (Figure S3A) (Gläscher et al., 2010). Reward payout explained independent BOLD fluctuations at feedback in dorsal putamen/insula, hippocampus, posterior cingulate cortex, and also a dorsal frontoparietal network (Figure S3B).

Motivated by an extensive literature implicating the dopamine-rich VM in updating beliefs (Klein-Flügge et al., 2011, Montague et al., 1996), we interrogated the BOLD response in VM (ROIs) (defined independently using coordinates from Klein-Flügge et al., 2011). We found that VM activity was best explained by a GLM that included both the stimulus-outcome update and the reward payout (unsigned stimulus-outcome update: *t*(21) = 2.39, p = 0.01; reward magnitude: *t*(21) = 2.01, p = 0.03; Figure 4B; see Experimental Procedures and GLM3; see Supplemental Information and Figures S4 and S5 for a related analysis of VM and whole-brain responses in terms of reward prediction errors). Moreover, those subjects in whom the reward payout (but not stimulus-outcome update) more strongly drove learning behaviorally showed stronger neural reward payout effects in VM (partial correlation between neural and behavioral reward payout effects [see Figure 2C], controlling for the behavioral effects of the previous S-O association and the S-O update: ρ = 0.63, p < 0.005; Figure 4B). This finding provides a direct link between the (inaccurate) influence of reward payout on updating behavior and VM neural response at choice feedback, yet it leaves open where in the brain these VM signals act to modify stimulus-outcome associations.

### lOFC and VM Feedback Responses Explain Single-Trial Change to Hippocampal CSS

**Figure 5:**
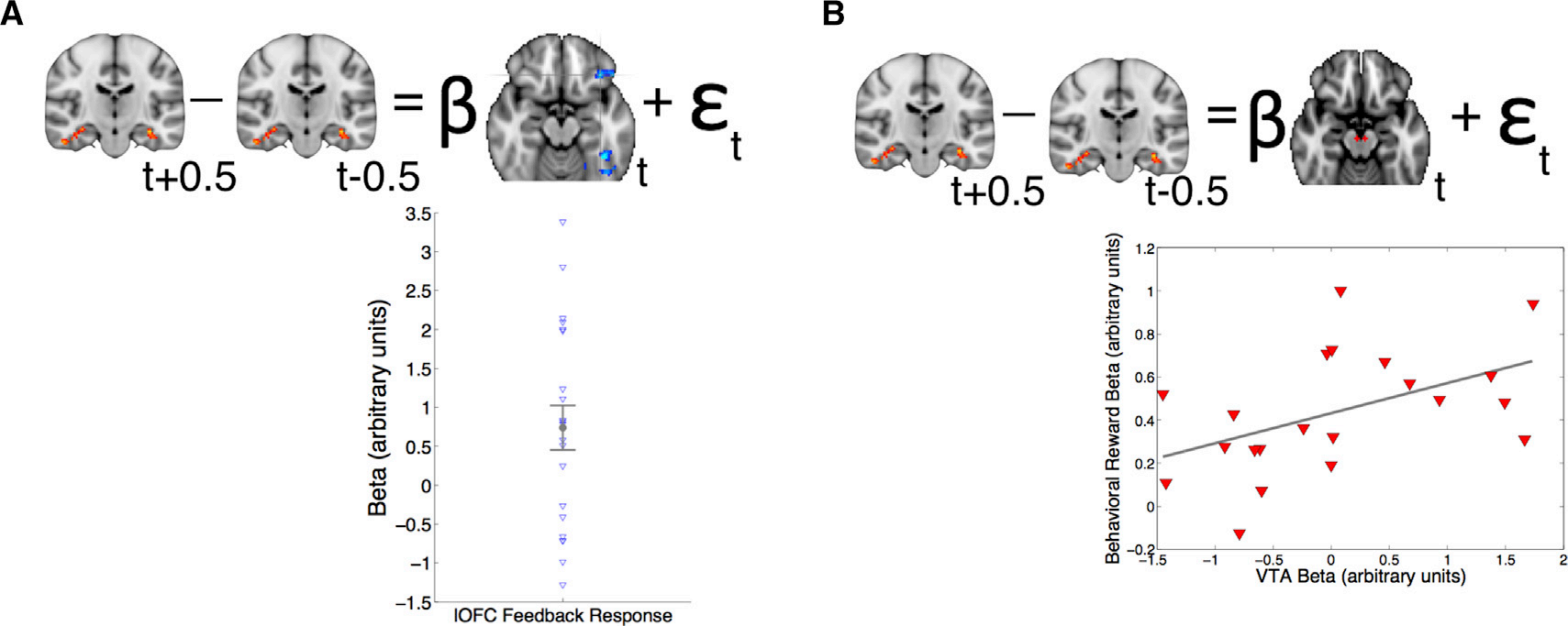
Feedback Activity during Choice Trials Predicts Changes to CSS Effects (A) Top: Depiction of multiple linear regression model (nuisance regressors not shown, see main text). Bottom: Mean ± SEM of group effect (β) shown in gray, and individual subjects, shown in blue, for lOFC feedback-locked signal (t(21) = 2.5, p = 0.01, one-sample t test). t + 0.5 refers to the suppression block after choice trial t and t − 0.5 to the block before. (B) Top: VM signal replaces lOFC in the regression model. Bottom: Scatterplot illustrates positive association across participants between the behavioral reward payout effect and the neural feedback-locked effect in VM on the single-trial change to hippocampal suppression during suppression blocks (partial Pearson’s correlation controlling for behavioral stimulus-outcome effect: ρ = 0.49, p = 0.02).

Analyses of feedback-related activity during choice trials identified S-O update effects in lOFC and both S-O update and reward effects in VM. We predicted these update signals might determine how much associations change as a result of the most recent choice feedback. CSS analyses, on the other hand, revealed flexible encoding of trial-by-trial associations in hippocampus and interconnected and surrounding regions. We sought to home in on the neural dynamics underlying learning by testing whether the feedback-locked signals could predict the change in hippocampal CSS as a result of single intervening choice trials. To test this prediction, we extracted the feedback-locked BOLD response in left lOFC (at 6 s post-feedback onset) at trial *t* and regressed this against the (signed) change to hippocampal CSS (i.e., the change in the difference between LC and HC presentations in [ipsilateral] left hippocampus from the preceding block *t* − 0.5 to the subsequent block *t* + 0.5) (Figure 5A; Experimental Procedures, GLM4). Note that the measurements of independent and dependent variables for this analysis were made at different times in the experiment: at choice feedback and item presentation during CSS blocks. We also included the local hippocampal feedback response, the model-estimated stimulus-outcome update, and the reward payout as nuisance regressors to test whether the lOFC feedback response explained the change to hippocampal CSS over and above these alternative variables (which did not significantly explain changes to hippocampal CSS). This analysis revealed a significant positive effect of the lOFC feedback response (*t*(21) = 2.50, p = 0.01; Figure 5A), indicating that fluctuations in lOFC responses at choice feedback predicted changes to the difference in hippocampal responses to LC and HC item presentations.

Although our a priori hypotheses focused on interactions between lOFC and hippocampus, based on the co-activation of these structures when predicting outcome identities in previous studies (Howard et al., 2015, Klein-Flügge et al., 2013), we also performed post hoc tests using each region that showed effects of $D_{KL}$ at feedback (Table S2). For example, the feedback responses in dorsolateral frontal and posterior parietal cortical regions that showed effects of the unsigned $D_{KL}$ (Figure S3), and have previously been linked to state prediction errors (Gläscher et al., 2010), did not explain a significant amount of variance related to the change in hippocampal CSS (both p > 0.3). In addition, we tested whether the lOFC feedback response predicted changes to CSS effects in neighboring peaks in the medial temporal lobe, including in perirhinal cortex, which receives monosynaptic projections from OFC in macaques (Kondo et al., 2005), and provides a major neocortical input into hippocampus (Bird and Burgess, 2008) and amygdala, which is reciprocally connected to OFC in macaques (Carmichael and Price, 1995, Stefanacci and Amaral, 2002) and whose functional interactions with OFC have been the topic of active investigation across species (Hampton et al., 2007, Morrison et al., 2011, Rudebeck et al., 2013a, Stalnaker et al., 2007). These analyses revealed some evidence that lOFC feedback responses also explained changes to CSS in perirhinal cortex (*t*(21) = 1.51, p = 0.07) and amygdala (*t*(21) = 1.85, p = 0.04) ROIs that showed CSS group effects (Tables S1 and S2).

ROI analyses revealed update and reward effects in VM during choice trials, and the latter was encoded more strongly in those subjects whose learning behavior displayed a stronger reward payout effect. To ascertain whether these VM responses might likewise update hippocampal associations following especially influential rewards, we performed the same analysis as described above, replacing left lOFC with left VM as the independent variable. Although we did not find a significant group mean effect (p > 0.4), there was considerable inter-individual variability. We found that the degree to which reward payout (but not stimulus-outcome update) influenced behavior correlated positively with the degree to which VM feedback-locked responses explained the change to hippocampal CSS across participants (partial correlation: ρ = 0.49, p = 0.02; Figure 5B). This analysis demonstrates that fluctuations in VM feedback responses had a stronger relationship with subsequent hippocampal encoding of stimulus-outcome associations in those subjects whose behavior was more strongly and inaccurately influenced by reward payouts.

## Discussion

Flexible decision making in response to changeable internal states and external circumstances necessitates mechanisms for acquiring, storing, and deploying an internal model of the world that maps choices to potential outcomes. By probing and modifying associations as learning progresses, we have shown that BOLD suppression in hippocampus, amygdala, and surrounding association cortex tracks the degree of association between particular stimuli and particular outcome identities—a basic internal model. Feedback responses in lOFC (among other brain regions) reflected updating terms important for acquiring and revising beliefs about associations between stimulus choices and ensuing outcome identities, whereas responses in VM additionally reflected updating based on reward payouts. By isolating single updates to associations during learning, we could further show that the learning-related signals in lOFC and VM predicted the subsequent change to CSS measured in the hippocampus and other medial temporal lobe structures. Taken together, these findings suggest that lOFC and VM update beliefs about stimulus-outcome transitions flexibly stored on-line or indexed in hippocampus, amygdala, and surrounding higher-level sensory and association cortex.

Previous studies have pointed to lOFC involvement in learning and/or using choice-outcome associations to guide behavior (Buckley et al., 2009, Gremel and Costa, 2013, Jones et al., 2012, Noonan et al., 2011, Rudebeck and Murray, 2014, Rudebeck et al., 2013b, Rushworth et al., 2011, Takahashi et al., 2011, Walton et al., 2010, Wilson et al., 2014). In animal models, lesions to lateral portions of macaque OFC produce deficits in appropriate credit assignment, given the task structure—or the appropriate attribution of particular reward outcomes to particular past stimulus choices (Walton et al., 2010)—and OFC inactivation in rats causes abnormal dopaminergic reward prediction error signals that can be elegantly accounted for by the loss of choice memory necessary for appropriate credit assignment (Takahashi et al., 2011). In humans, lOFC BOLD responses are increased when stimulus-response associations are guided by consistent rather than inconsistent reward outcomes (Noonan et al., 2011) and show differential updating signals consistent with social credit assignment (Boorman et al., 2013a). Here, we isolate a particular computational role for lOFC in updating stimulus-outcome associations at choice feedback that may at least partly underpin its involvement in some of these previous studies. Notably, that lOFC signaled stimulus-outcome updates, but was not sensitive to the online stimulus-outcome association strength during CSS blocks, suggests that lOFC may utilize an internal model to update beliefs about, or facilitate decisions between, rewarding goals (Stalnaker et al., 2015, Wilson et al., 2014) but may not store the model locally. Importantly, the rostrolateral localization of the OFC update signal in our study can be contrasted with signals in more medial subdivisions of OFC, and neighboring subdivision within ventromedial PFC, that have been shown to encode abstract values and comparisons between goal values during choices (Boorman et al., 2009, Boorman et al., 2013b, Chib et al., 2009, Howard et al., 2015, Lim et al., 2011).

lOFC, extending into VLPFC, was one region in a network that included ACC, inferior temporal gyrus, and posterior cingulate cortex, which all showed significant effects of the *D*_*KL*_, a measure of the information contained in the belief update at choice feedback. Notably, these signals were signed based on the subject’s goal, consistent with a mechanism for determining how much to update beliefs and in which direction: toward confirmation (positive) or reconsideration of one’s rewarding goal (negative). Importantly, unlike the VM signal, the lOFC signal on average was not additionally sensitive to the reward payout obtained and so is distinct from a reward prediction error. ACC recruitment is consonant with demonstrations that belief updating signals can be measured in a slightly more dorsal ACC sulcal subdivision in the context of instrumental reward value learning (Behrens et al., 2007) and perceptual choice (O’Reilly et al., 2013), complementing evidence that lesions to this region in macaques produce impairments in the appropriate integration of past reward (Kennerley et al., 2006). Update effects in inferior temporal gyrus, an area sensitive to the abstract visual stimuli between which participants selected, may reflect reactivation of the relevant stimulus representation in order to update the appropriate association.

The network encoding signed *D*_*KL*_ can be contrasted with a dorsal frontoparietal network that reflected the unsigned *D*_*KL*_, consistent with a previous demonstration that unsigned state prediction errors, signaling errors in probabilistic transitions between states during latent learning in the absence of reward, recruited a similar network (Gläscher et al., 2010). It remains unclear whether the distinction between these two networks depends on learning about stimulus–reward outcome transitions, a subset of state–state transitions, or instead how the update signals are signed, here with respect to the subject’s reward goal, which was notably absent from the latent learning blocks in which state prediction errors were previously measured. Notably, unlike lOFC, the feedback response in these frontoparietal areas did not predict the subsequent change to hippocampal suppression (all p > 0.3). Although it is unclear precisely what the contribution of this dorsal frontoparietal network is, it may nevertheless play a key role in updating such internal models.

Interrogation of the BOLD response in VM revealed effects of both unsigned stimulus-outcome updates and reward payouts. Intriguingly, the update effect fits nicely with recent reports of unsigned precision-weighted prediction errors in VM during an auditory-visual learning task, where learning was orthogonal to reward (Iglesias et al., 2013), and similar measures of belief updating (*D*_*KL*_), but not information-theoretic surprise, about the relevance of an auditory or visual stimulus modality in predicting reward (Schwartenbeck et al., 2016). Our results further show that when learning is inaccurately influenced by reward, VM BOLD activity is additionally sensitive to the reward outcomes at choice feedback. Because reward payouts for the two outcomes were inversely correlated in our task, this unsigned signal may reflect reward-based updating of the best choice or policy, given the outcome obtained: the selected option following preferred outcomes and the counterfactual option following unpreferred outcomes. Alternatively, it may reflect the extent to which salient outcomes lead to shifts in beliefs. Further studies are necessary to experimentally tease apart these and other possibilities. In either case, the reward effect in VM was tightly coupled to the influence of reward payout on learning behavior.

Motivated by recent demonstrations that presentation of pairs of stimuli, or stimuli and reward outcomes, which have previously been associated deterministically, lead to a reduction in both neuronal and BOLD responses when compared to unassociated items (Barron et al., 2013, Klein-Flügge et al., 2013, Meyer and Olson, 2011), we hypothesized we could exploit CSS to probe the degree of association acquired on-line during choice trials, circumventing potential confounds present during choice and updating. This approach revealed that the BOLD response in a network including hippocampus, parahippocampal gyrus, amygdala, perirhinal cortex, inferior/middle temporal gyrus, temporal parietal junction area, and posterior cingulate cortex suppressed in proportion to the association strength, estimated using a Bayesian reversal-learning model. This analysis demonstrates that activity in these regions was sensitive to the on-line association strength between stimuli and outcomes, flexibly acquired, and updated during learning, consistent with the flexible encoding of a basic internal model.

Different mechanistic accounts have been advanced to explain RS, including fatigue, sparse coding, and predictive coding (Grill-Spector et al., 2006, Summerfield et al., 2008, Wiggs and Martin, 1998). Although there is not yet consensus on the underlying mechanism, our controlled analysis, comparing the same outcome when it was preceded by a more or less associated stimulus, means that the only difference between items was the association with the preceding stimulus, acquired from choice trials. Plausible mechanisms underlying the relationship we observed between suppression and association strength include predictive coding of outcomes elicited by stimulus presentation (Summerfield and Egner, 2009) and/or plasticity between the underlying neuronal populations that encode a particular stimulus and a particular outcome, which become increasingly overlapping with learning. In either case, because the association strength was de-correlated from the likelihood that a particular stimulus or outcome would be presented during CSS blocks, the suppression measured must be related to the association acquired during choice trials, rather than the statistical sequence of items presented during probe blocks. It is possible that such CSS measures would also be sensitive to the statistical transitions observed during CSS blocks. However, because each possible pairing was presented with equal frequency during each CSS block, this meant that any incidental learning about S-O transitions should equate on average, thereby obviating any attempt to detect this in our paradigm. It will be important to establish the extent to which the CSS measure is sensitive to such incidental learning in future experiments.

It can be informative to compare the identification of this medial temporal lobe network in flexibly encoding the online, stochastic relationship between particular predictive stimuli and reward outcomes with other recent findings on stimulus-outcome associations in the literature. In particular, studies using simpler prediction tasks involving deterministic and well-learned pairings between stimuli and reward outcomes have found the encoding of stimulus-outcome associations in rostrolateral OFC, and outcome identity or attribute coding, independently of the predictive stimulus, in hippocampus and caudolateral OFC (Klein-Flügge et al., 2013) or hippocampus and more rostrolateral OFC (Howard et al., 2015). Two potentially important differences between our study and these previous ones concern the statistical and labile nature of the associations between stimuli and outcomes used here, which were stochastic and had to be updated flexibly throughout the experiment, as opposed to the deterministic and well-learned associations used in these previous studies. Understanding precisely when and how the lOFC and hippocampus contribute to storing or using stimulus-outcome associations and expectations about outcome identities or attributes, both important for generating internal models of the world or a “task space,” is an important question to address in future studies.

To test whether computational learning signals measured in one region can impact on task representations measured in another, we isolated single-trial changes to the CSS index of association strength in hippocampus and tested whether neural responses in lOFC and VM at choice feedback predicted these changes. The feedback-related lOFC response predicted fluctuations in the single-trial change to hippocampal CSS across all participants, while VM did so to the extent that reward payout inaccurately shifted participants’ beliefs. Importantly, these effects remained significant after including the model-derived update and reward terms and local hippocampal feedback response in the regression model, indicating that residual feedback-related activity in these structures explained variance in the changes to hippocampal CSS over and above these additional terms. Although we cannot infer causality from this analysis, it implies that lOFC and VM updating during choice feedback shapes the encoding of associations between particular items in hippocampus and surrounding medial temporal regions. Such long-range functional interactions could only be interrogated through the combination of a whole-brain imaging technology and a technique to probe representation as it evolves during learning.

lOFC interactions with hippocampus may stem from indirect connections via interconnected perirhinal cortex, which receives monosynaptic connections from OFC (Kondo et al., 2005). Notably, disconnection of rhinal cortex and orbital frontal cortex in macaques leads to impairments in learning visual stimulus to reward associations (Clark et al., 2013), although the underlying mechanism has been unclear. This possibility is hinted at by the marginal effect of lOFC feedback responses on the change to perirhinal CSS. It should be noted, however, that the signal dropout and distortion around this very anterior and ventral cortical region mean that the data are inherently less robust. Intriguingly, post hoc tests also revealed a correlation between lOFC feedback activity and the single-trial change to CSS in amygdala, complementing and extending previous demonstrations these interconnected structures interact during learning (Morrison et al., 2011, Stalnaker et al., 2007).

While most previous research has focused on hippocampal interactions with medial prefrontal cortex, notably when associative information is used to evaluate or imagine choices online (Barron et al., 2013, Kumaran et al., 2009, Peters and Büchel, 2010, Wikenheiser and Redish, 2015), lOFC-hippocampal interactions have been relatively unexplored. Our results suggest they also play a key role in the context of goal-directed control. DA neurons in VM, on the other hand, have direct projections to hippocampus proper (Gasbarri et al., 1994), and learning-related coupling between these structures has previously been discovered in the context of facilitating generalization and long-term memory formation (Shohamy and Wagner, 2008, Wittmann et al., 2005). Our findings suggest this relationship extends to the influence of reward-based updating on the learning of task structure during decision making.

We have advanced an account of how learning-related signals impact neural representations of associations between crucial task variables in distant structures. An important extension of this work concerns how these associations are then leveraged to flexibly construct subjective goal values for particular outcomes that guide flexible choices (Hare et al., 2008, Jones et al., 2012, Wunderlich et al., 2012). The methodological approach we present here holds promise to probe the dynamics of such representational questions during learning and choice.

## Experimental Procedures

### Participants

Twenty-six healthy human volunteers participated in the fMRI experiment. Four participants were excluded because they failed to reach our threshold criterion of ≥75% correct performance during the incidental 1-back task during CSS blocks, resulting in 22 participants included in all subsequent behavioral and neural analyses. We introduced this criterion because we required assurance that participants attended to each item presented during CSS blocks. The sample size was based on similar sample sizes in recent fMRI studies of decision making. Participant identities were anonymized for analyses. Participants were aged 22 to 33 (mean age: 25.82), 11 were female, and 18 were right-handed. We excluded volunteers who had a history of any psychiatric or neurological condition or those who were on psychotropic medication. The study was approved by a local University of Oxford ethics committee (ref: MSD-IDREC-C1-2013-066), and all participants gave written informed consent.

### Experimental Task

Participants first rated each of six gift cards from 1 (minimum desirability) to 100 (maximum desirability) using a track ball. We selected the two gift cards that were maximally rated, to ensure gift card outcomes were incentivizing in the fMRI task. Participants then all passed an experimental quiz testing key concepts about our task, such as full dependence between selected and unselected stimuli and the ensuing outcomes and the irrelevance of reward payouts but not stimulus-outcome associations for future behavior. During training, participants learned associations between different stimuli and gift cards from those used in the fMRI task, using a random schedule of stimulus-outcome transitions and different random payouts (sampled independently on each trial from a uniform distribution between 1 and 100). A few training trials were also conducted in the scanner to familiarize participants with the button box.

For the fMRI experiment, we counterbalanced the assignment of particular stimuli to a schedule of gift card outcomes and reward payouts across participants. This procedure meant that the particular identity of the HC and LC pairs at any trial during the experiment was reversed for half of the subject sample. Participants were informed that one choice trial would be selected at random at the end of the experiment and that this would constitute their actual payout on the gift card obtained on that trial, so it was advantageous for them to treat each choice as if it counted “for real.” At the end of the experiment, we randomly selected one trial and divided the points obtained on that outcome by three (we repeated this procedure if this would have resulted in payment less than £10, but this was not known to participants). This procedure yielded a mean payout of £20.76 on a gift card, which we rounded to the nearest £5 mark. As shown in Figure 2, the true stimulus-outcome probabilities changed such that the identity of the more likely outcome reversed at trial 26, while two new stimuli were introduced at trial 51. The motivation for including new stimuli at trial 51 was to test whether there would be any differences between the neural CSS effects when subjects modified or reversed a learned S-O association and when they learned a new S-O association. No such differences in neural effects were observed, even at a reduced threshold of p < 0.05 uncorrected, so we treated these phases identically in our subsequent neural analyses. In total, this constituted 75 choice trials.

In choice trials, participants saw two abstract stimuli, each randomly presented on either the left or right side of the screen, and two numbers that summed to 100. One of these numbers n1 was sampled independently on each trial from a uniform distribution between 1 and 100, and the other n2 was defined as 100 − n1. A number’s color indicated with which gift card it was deterministically paired. These numbers represented potential payouts that could be won on the gift cards, if obtained. Their position on the screen, either at the very top or just beneath, was determined randomly on each trial. After a jittered interval, a question mark appeared that served as a go cue, after which participants had to select a response with a button press, mapped to the location of the stimulus on the screen, within 3 s or else the trial aborted. The selected option was then highlighted for 0.5 s, followed by presentation of the gift card outcome, and associated payout for another jittered interval. These jittered choice and outcome periods facilitated dissociation of these events in time for fMRI analyses, yet they precluded meaningful analyses of behavioral reaction times.

With independently drawn transition probability P, choice of stimulus 1 led to gift card 1, and with probability 1 − P, to gift card 2. The inverse relationship governed the transitions between stimulus 2 and gift cards 1 and 2 (see Figures 1 and 2). A single schedule of transition probabilities and reward payouts was selected to de-correlate key variables of interest, and this schedule was used for each participant (Figure S1). Importantly, subjects did not know the true underlying reward probabilities, or true reversal probability, but had to learn these model parameters through trial-and-error feedback.

Each choice trial was followed by a jittered ITI before presentation of the first stimulus of the next CSS block. In CSS blocks, stimuli, and outcomes (nine items per block) were presented in a pseudorandom and interleaved sequence, ensuring that each stimulus-outcome transition and each outcome-stimulus transition was presented twice per block. These CSS blocks were presented after the first choice trial and each choice trial thereafter, totaling 75 CSS blocks. Incidental catch trials were presented once per CSS block on average and could be presented at any position in the sequence of nine items. In addition to the reward payout on gift cards, participants were endowed with £25 from which £1 was deducted for incorrect responses during the incidental task in CSS blocks, resulting in a mean payment of £19.14 (SD = £4.78). On average, the subjects correctly identified 69.14 (SD = 4.78) out of 75 catch trials. For these incidental trials, all four items (both stimuli and both gift cards) were presented at random locations, and participants had to press a button corresponding to the location of the last item presented. Feedback was only delivered for incorrect responses, which informed participants they had lost £1 from their endowment. Incorrect CSS items were excluded from fMRI analyses.

### Behavior

In order to generate behavioral and neural predictions, we constructed a Bayesian reversal learning model (Table 1; Supplemental Information) that reflected the information communicated to participants—in particular, that true transition probabilities between stimuli and outcome identities were inversely related and that the identity of the more likely outcome following choice of a particular stimulus might reverse unexpectedly. See Table 1 and Supplemental Experimental Procedures for details of model fit and behavioral regression analyses, including GLM1.

### fMRI

fMRI data acquisition, preprocessing, and ROI analyses are described in detail in the Supplemental Experimental Procedures.

### Genera Linear Model Estimation

Separate GLMs were fit in pre-whitened data space to identify stimulus-outcome updating during choice feedback and association encoding during CSS blocks (Woolrich et al., 2001). All regressors were convolved with FSL’s canonical gamma hemodynamic response function and temporally filtered with the same high-pass filter applied to the fMRI time series.

We computed GLM2 to probe associations during CSS blocks. We defined separate explanatory variables (EVs) for each individual gift card outcome during CSS blocks (300 total EVs). For GLM2, we then defined the following contrasts of parameter estimates (COPEs):

(1) LC-HC item events, classified as LC or HC based on the Bayesian model’s current estimate of the mean transition probabilities *r*_*t*_ and 1 − *r*_*t*_, having witnessed the most recent choice feedback at trial *t* − 0.5. This COPE is shown in Figure S3.
(2) The difference between LC and HC item events defined above, modulated by the trial-by-trial difference in association strength between HC and LC items: *r*_*tHC*_ − *r*_*tLC*_. In other words, this COPE tested for a difference between LC and HC item presentations that was proportionate to the difference between model estimates of HC and LC transition probabilities. This COPE is shown in Figure 3.

We defined a separate GLM3 to identify learning-related update effects at choice feedback. Specifically, for GLM3, we divided choice outcomes into preferred (or more expected/common) and non-preferred (or less expected/rare) transitions, based on our definition of $o_{p}$ (see Equation 12 in Supplemental Information) and modulated these different outcomes by the stimulus-outcome update $D_{KL}$ and reward payout sizes *m*:

GLM3

$$Y=\beta_{0}+\beta_{1}i_{p}+\beta_{2}i_{np}+\beta_{3}i_{p}D_{KL}+\beta_{4}i_{np}D_{KL}+\beta_{5}i_{p}m_{b-w}+\beta_{6}i_{np}m_{b-w}+\beta_{7}DEC+\varepsilon ,$$

where $i_{p}$ = 1 if the preferred outcome $o_{p}$ is obtained and 0 otherwise and $i_{np}$ = 1 if the non-preferred outcome $o_{np}$ is obtained and 0 otherwise. The duration of these feedback events corresponded to their true duration in the experiment (2–4 s jittered across trials). The term $m_{b-w}$ denotes the difference between reward magnitudes for best and worst outcomes, once again defined using individual indifference points. The final term *DEC* refers to the main effect of the decision event, with duration 2.5–5.5 s (jittered across trials) + RT. Using this GLM, we then defined COPEs for signed *D*_*KL*_ as *β*_3_ − *β*_4_ and unsigned *D*_*KL*_ as *β*_3_ + *β*_4_. Note that because preferred and non-preferred outcomes were modeled separately, any effects of signed or unsigned *D*_*KL*_ cannot simply be explained by differences between preferred and non-preferred outcomes (see Figure S2 for the z-statistic map pertaining to preferred versus non-preferred binary outcomes). We also defined reward for best relative to worst outcomes as *β*_5_ + *β*_6_. GLM3 was used to produce the Z-stat map in Figure 4A and the time course plots from lOFC and VM in Figures 4A and 4B. For GLM3 (but not for GLM2 due to event timings), temporal derivatives of all regressors were also included to account for variability in the hemodynamic response function.

For GLM4, we tested whether the feedback-related BOLD response (6 s post-feedback onset) at trial *t* in lOFC, VM, DLPFC, or posterior parietal cortex ROIs predicted the single-trial change in suppression between LC and HC items from the preceding CSS block at trial *t* − 0.5 to the subsequent CSS block at trial *t* + 0.5:

GLM4

$$\Delta Y_{LC-HC}=\beta_{0}+\beta_{1}ROI_{6s}+\beta_{2}ROI_{HC_{6s}}+\beta_{3}i_{p}D_{KL}+\beta_{4}i_{np}D_{KL}+\beta_{5}i_{p}m_{b-w}+\beta_{5}i_{np}m_{b-w}+\varepsilon ,$$

where $\Delta Y_{LC-HC}$ denotes the change in the difference between LC and HC items from one block to the next, ${\left(\text{LC}-\text{HC}\right)}_{t+0.5}-{\left(\text{LC}-\text{HC}\right)}_{t-0.5}$, and $ROI_{6s}$ and $ROI_{HC_{6s}}$ denote the BOLD response at 6 s post-feedback from trial *t* in the ROI tested (either lOFC or VM, shown in Figure 5) and also for the nuisance regressor in hippocampus, respectively.

### Second-Level GLM and Statistical Inference

For group analyses, we fit a GLM to estimate the group mean effects for the regressors described above. Ordinary Least-squares in FEAT was used to perform a mixed effects group analysis. To detect and de-weight outliers, we performed robust group analysis using outlier inference, applying FEAT’s outlier de-weighting option (Woolrich, 2008). All reported fMRI Z-statistics and p values arose from these mixed effects analyses on all 22 subjects. Unless otherwise stated, we report significant effects at a cluster-forming threshold across the whole brain of Z > 2.3 and a family-wise error rate of p = 0.05.

## Author Contributions

E.D.B. and T.E.B. designed the experiment; E.D.B. and V.G.R. collected data; E.D.B., V.G.R., J.X.O.’R., and T.E.B. performed analyses; and E.D.B., J.X.O.’R., and T.E.B. wrote the paper.

## Figures and Tables

**Table 1:** Model Comparison of Behavior

Model	Parameters (per subject)	α	τ	η	NLogL (sum)	BIC (sum)
Reversal Model	2	0.94	0.18	NA	555.02	1,448.4
Experience-Weighted Reversal Model	3	1.03	0.20	1.01	537.07	1,589.7
Volatility Model	2	0.93	0.15	NA	636.43	1,611.2

A comparison of Bayesian reversal, experience-weighted reversal, and volatility models, including the number of parameters in the model (per subject), the subject mean maximum likelihood estimate for terms in the models, the negative log likelihoods (summed over participants), and the Bayesian Information Criterion (summed over participants). α denotes the outcome magnitude-weighting term; τ denotes the choice sensitivity parameter; η denotes the experience/inferred weighting term; NlogL denotes negative log likelihood; BIC denotes Bayesian information criterion. Lower NlogL and BIC values indicate better fits to behavior.

## Supplemental Information

Document S1. Figures S1–S5, Tables S1 and S2, and Supplemental Experimental Procedures

Document S2. Article plus Supplemental Information
