# Supplementary material for: Two Anatomically and Computationally Distinct Learning Signals Predict Changes to Stimulus-Outcome Associations in Hippocampus
Source: Neuron. 2016 Mar 16;89(6):1343–54. doi: 10.1016/j.neuron.2016.02.014 (PMC4819449; doi:10.1016/j.neuron.2016.02.014)
Supplement: Document S1. Figures S1–S5, Tables S1 and S2, and Supplemental Experimental Procedures [file mmc1.pdf]

Neuron, Volume *89*

Supplemental Information

Two Anatomically and Computationally Distinct Learning Signals Predict Changes to Stimulus-Outcome Associations in Hippocampus

Erie D. Boorman, Vani G. Rajendran, Jill X. O'Reilly, and Tim E. Behrens

Supplemental Information

Supplemental Experimental Procedures

Bayesian reversal learning model

Structure of the learning task

On each trial, participants selected one of two stimuli {S_1, S_2} and observed one of two outcomes {O_1, O_2}. Since participants were informed that each stimulus was associated with exactly one outcome on each trial and vice versa, this single observation gave full information about the stimulus-outcome contingencies on the current trial:

$$(S_1 \rightarrow O_1) \Rightarrow (S_2 \rightarrow O_2)$$

Eq. 1

Hence estimating the contingency between once S-O pair $p(S_1 \rightarrow O_1)$ is equivalent to estimating the full contingency structure. Let the true probability that S_1 leads to O_1 on trial *t*, $p_t(S_1 \rightarrow O_1)$, be denoted by q_t. Then:

$$p_t(S_1 \rightarrow O_1) = q_t$$
$$p_t(S_2 \rightarrow O_2) = q_t$$
$$p_t(S_1 \rightarrow O_2) = 1 - q_t$$
$$p_t(S_2 \rightarrow O_1) = 1 - q_t$$

Eq. 2

The true value of q_t was in fact 0.75 in the first 25 trials and either 0.8 or 0.2 in the next 50 trials. These values were not known by participants. Participants were instructed that the contingencies could reverse but were not told when. In reality when the contingencies did reverse the first time, their new true values changed slightly from 0.75/0.25 to 0.8/0.2. These values were chosen simply because they proved to be effective in a previous study[1].

Let the presence of a reversal on trial t be denoted by J_t such that

$$J_t = \begin{cases} 1 \text{ if there is a reversal on trial t} \\ 0 \text{ otherwise} \end{cases}$$

Eq. 3

then

$$q_t = q_{t-1} \cdot \delta(J_t, 0) + (1 - q_{t-1}) \cdot \delta(J_t, 1)$$

Eq. 4

where δ denotes the Kroenecker delta function.

Participants were not instructed as to the probability of reversal; in fact the contingencies reversed once after 25 trials.

After 50 trials the stimuli {S_1, S_2} were replaced with a new pair {S_3, S_4} at which point the probabilities $p_t(S_3 \rightarrow O_1)$ had to be estimated afresh. The motivation for including new stimuli at trial 51 was to test whether there would be any differences between the neural CSS effects when subjects reversed a learned S-O association and when they learn a new S-O association. No such differences in neural effects were observed, even at the reduced threshold of $p<0.05$ uncorrected, so we treated these phases identically in our subsequent neural analyses.

Learning model

We constructed a normative Bayesian learning model that estimated the contingency q_t on each trial based on the history of observed outcomes, selected stimuli, and observed outcomes on trials up to and including trial *t,* denoted by $\boldsymbol{y_{1:t}}$.

On each trial *t,* the posterior probability for each value of q_t was given using Bayes' rule:

$$p(q_t|\boldsymbol{y_{1:t}}) \propto p(\boldsymbol{y_t}|q_t) \cdot p(q_t|\boldsymbol{y_{1:t-1}}, v)$$

Eq. 5

The likelihood $p(\boldsymbol{y_t}|q_t)$ is simply q_t.

The prior $p(q_t|\boldsymbol{y_{1:t-1}})$ accounts for the possibility of a reversal *J.* The probability of a reversal $v = p(J_t=1)$ was modeled as fixed across trials but of unknown value. Hence the prior $p(q_t|\boldsymbol{y_{1:t-1}})$ on trial *t* was obtained from the posterior on the previous trial by applying a transition function:

$$p(q_t|y_{1:t-1}) = \int \; [p(q_{t-1}|y_{1:t-1}) \cdot (1-v)] + [(1-p(q_t|y_{1:t-1})) \cdot v] \; dv$$

Eq. 6

When the stimuli {S_1, S_2} were replaced with a new pair {S_3, S_4} on trial 51, the learning model assumed that *v* was unchanged. Furthermore the model assumed that the stimulus-outcome contingencies were transferred to the new stimuli, such that either

$$p_{51}(S_3 \rightarrow O_1) = p_{50}(S_1 \rightarrow O_1)$$
or
$$p_{51}(S_4 \rightarrow O_1) = p_{50}(S_1 \rightarrow O_1)$$

Eq. 7

This decision was taken because it is a natural choice given the task instructions and because it slightly improved behavioral fits when compared with a variant of this reversal model that learned the stimulus-outcome contingencies anew. Let the new contingency $p_{51}(S_3 \rightarrow O_1)$ be denoted by q^*. Then the prior on trial 50, the first trial with the new stimuli was given by

$$p(q^*{}_{51}|\boldsymbol{y}_{\boldsymbol{1:50}}) = \tfrac{1}{2}p(q_{51}|\boldsymbol{y}_{\boldsymbol{1:50}}) + \tfrac{1}{2}p(1 - q_{51}|\boldsymbol{y}_{\boldsymbol{1:50}})$$

Eq. 8

For simplicity, let r denote the mean of the belief distribution over transition probabilities, given the past choice outcomes observed up to trial t: mean[$p(q_t|\boldsymbol{y}_{\boldsymbol{1:t-1}})$]. We used these normative estimates of transition probabilities to generate estimates of each participant's subjective expected value for a given stimulus 1 (S1):

$$g_{s1} = r_{S1\to O1}m_{o1}\alpha + r_{S1\to O2}m_{o2}\frac{1}{\alpha}$$

Eq. 9

where g_{s1} denotes the subjective expected value for stimulus 1, $r_{S1\to O1}$ denotes the belief in the transition probability from stimulus 1 to outcome identity 1, and m_{o1} denotes the reward payout on a particular gift card outcome. In our formulation, α is a subject-specific free parameter that allows for the possibility that participants weight reward payouts for one gift card more or less than reward payouts on the alternative gift card, indicative of differential preferences between gift cards. It follows that the subject expected value of the alternative stimulus 2 is given by:

$$g_{s2} = r_{S2\to O1}m_{o1}\alpha + r_{S2\to O2}m_{o2}\frac{1}{\alpha}.$$

Eq. 10

We assumed participants then selected between stimuli based on the following softmax distribution:

$$P(s) = \frac{\exp(\tau g_s)}{\sum_{s'=1}^{N_s}\exp(\tau g_{s'})}$$

Eq. 11

where τ is a second subject-specific free parameter that reflects the sensitivity of stimulus choices to expected stimulus values and N_s=2.

The experience-weighted Bayesian reversal learning model was identical to the above, except it contained an additional free parameter η that differentially weighted outcomes depending on whether they were experienced or inferred.

We fitted α, τ, and where applicable η to each individual subject's choices using standard non-linear minimization procedures implemented in MATLAB 14a (Mathworks). Based on these estimates, we inferred each subject's preferred gift card outcome O_p on each choice trial as the outcome with the greater magnitude, after weighting by α:

$$O_p = \max(m_{o1}\alpha, m_{o2}\frac{1}{\alpha}).$$

Eq. 12

We next tested to what extent estimates of association strength, updates to those associations, derived from the Bayesian reversal learning model, and the reward payouts obtained, captured fluctuations in participants' actual choices. In particular, we computed a linear regression model, predicting stimulus 1 choice on trial t on the basis of three terms:

$$c = \beta_1 r_{S1\rightarrow O_{p(t)},t-1} + \beta_2 \theta_{t-1\rightarrow t} + \beta_3 m_{o_{t-1\rightarrow t}} i_t, \qquad \text{GLM 1}$$

where $r_{S1\rightarrow O_{p(t),t-1}}$ is the *previous* belief that selecting stimulus 1 would lead to the *currently* preferred outcome, before seeing the latest outcome, $\theta_{t-1\rightarrow t} = r_{S1\rightarrow O_{p(t)},t} - r_{S1\rightarrow O_{p(t)},t-1}$, or the update to this association from the latest outcome, $m_{o_{t-1\rightarrow t}}$ denotes the latest reward payout obtained (specifically the amount of points obtained on the latest choice outcome) and i_t is an indicator term which determines the association that the reward payout 'stamps in':

$i_t = 1$ if selecting stimulus 1 on trial t-1 led to the *currently* preferred outcome O_p on the last outcome or stimulus 2 led to the *currently* non-preferred outcome O_{np} on the last outcome
$i_t = -1$ otherwise.

To define fMRI regressors to capture identity updating for fMRI analyses, we defined the stimulus-outcome updates as the Kullback-Liebler divergence between posterior and prior distributions over possible transition probabilities:

$$D_{KL}(t) = \int \ln\left(\frac{p(q_t|y_{1:t})}{p(q_t|y_{1:t-1})}\right) p(q_t|y_{1:t}) \quad dq$$

Eq. 13

MATLAB code for models is available on request.

FMRI Analyses

FMRI data acquisition, preprocessing, and analysis

FMRI data were acquired on a 3T Siemens TRIO scanner with a voxel resolution of 3x3x3 mm^3, TR=3s, TE=30ms, Flip angle=87°. The slice angle was set to 30° and a local z-shim was applied around the orbitofrontal cortex to minimize signal dropout in this region(Deichmann et al., 2003), which has previously been implicated in other aspects of learning and decision making. The mean number of volumes acquired was ~1034, giving a mean total experiment time of approximately ~52 minutes.

We acquired Field Maps using a dual echo 2D gradient echo sequence with echos at 5.19 and 7.65 ms, and repetition time of 444ms. Data were acquired on a 64x64x40 grid, with a voxel resolution of 3mm isotropic. T1-weighted structural images were acquired for subject alignment using an MPRAGE sequence with the following parameters: Voxel resolution 1x1x1 mm^3

on a 176x192x192 grid, Echo time(TE)= 4.53 ms, Inversion time(TI)= 900 ms, Repetition time (TR)= 2200 ms.

Preprocessing and analysis of fMRI data was performed using tools from FEAT (fMRI Expert Analysis Tool) Version 6.00, part of FSL (FMRIB's Software Library, http://www.fmrib.ox.ac.uk/fsl)[52]. Region-of-interest time series analysis was performed using custom-written scripts in MATLAB 14a (Mathworks). Data were preprocessed using the default options in FEAT: motion correction was applied using rigid body registration to the central volume(Jenkinson et al., 2002); corrected for geometric distortions using the field maps and an n-dimensional phase-unwrapping algorithm(Jenkinson, 2003); Gaussian spatial smoothing was applied with a full width half maximum of 5mm; brain matter was segmented from non-brain using a mesh deformation approach; high pass temporal filtering was applied using a Gaussian-weighted running lines filter, with a 3dB cutoff of 100s; and slice timing correction for ascending interleaved sequence was applied. EPI images were registered with the high-resolution structural images and normalized into standard (MNI) space using affine registration using FLIRT (FMRIB's Linear Image Registration Tool)(Jenkinson and Smith, 2001).

Region of interest analysis

Time series for ROI plotting and analyses were determined by generating a 3mm radius sphere in standard space centered on coordinates from previous studies: ref (Klein-Flugge et al., 2013) for lOFC and ref (Klein-Flugge et al., 2011) for VM. We then applied the inverse of each individual's registration, calculated during intersubject registration, to project this mask from standard space to the 3-mm^3 isotropic space in which EPI data were acquired and extracted the mean time series within this region of interest from the pre-processed EPI data for each subject.

To plot effects of individual regressors through time, the timeseries was upsampled, then time-locked to feedback onset (Figure 4) of each trial. This creates a data matrix with dimensions nTrials*nTime points within a trial. Each time point was regressed against explanatory variables of interest for each subject. The mean ± standard error (across subjects) of parameter estimates from this regression is plotted. A full description of this approach is given in ref (Behrens et al., 2008).

To obviate the potential for selection bias when conducting statistical tests reported in the section *"lOFC and VM feedback responses explain single-trial change to hippocampal CSS"* and Figure 5, we adopted a leave-one-out approach to ROI construction, in which the lOFC and hippocampal masks used to extract each subject's data were based upon coordinates from a group analysis containing all the remaining ($n - 1$) subjects, and then tested in the independent left-out subject.

Supplemental Data

Behavioral Model Comparison

As stated in the main text, the purpose of the Bayesian reversal learning model was to generate trial-by-trial predictions to relate to neural responses, rather than to optimally capture behavior and as such, we did not compare an extensive range of models. However, to test whether the model outperformed an alternative, well-established Bayesian model in the context of our task, we compared performance with a previously described hierarchical Bayesian learning model ("Volatility Model", see ref[1] for a detailed description). Briefly, the volatility model contains a belief volatility term that controls the rate of change of the outcome probability, and an additional hyperparameter that represents the distrust in the constancy of the volatility. The model effectively assumes that unlikely outcomes lead to Gaussian-governed drifts in the outcome probability estimate, controlled by the estimate of the environmental volatility, rather than to potential reversals. As shown in table S1, the Bayesian reversal-learning model we constructed for the current task more accurately captures participants' choices in our task.

Reward prediction error analyses

We computed a separate GLM in a whole-brain analysis in which we modulated feedback events by reward prediction errors, defined as the reward amount obtained minus the subjective expected value:

$$\delta = m_o - g_{s_{ch}},$$

where $g_{s_{ch}}$ is the subjective expected value for the chosen stimulus. This analysis revealed several clusters, including in ventral striatum (peaks in nucleus accumbens and subgenual cingulate), left hippocampus, and sensorimotor cortex (Figure S5).

In addition, we conducted an alternative analysis of feedback-locked activity in VTA ROIs in terms of reward prediction errors (rPEs). We found that VTA activity was consistent with an unsigned rPE, generated using estimates of the transition probabilities and potential reward payouts ($t(21)=2.81$, $p=0.005$). These effects thus depended on the preferred outcome: VTA signaled positive rPEs for preferred outcomes and negative rPEs for unpreferred ones (Figure S4). Moreover, those subjects in whom the reward payout (but not identity update) more strongly drove learning behaviorally showed stronger unsigned reward prediction error effects (partial correlation $\rho = 0.50$, $p = 0.025$; Figure S4). We note that this formulation is closely related, but not identical, to the model (GLM3) presented in the main text that uses a combination of D_{KL} and reward payout to explain fluctuations in VTA responses. We elect to present the results of GLM3 in the main text because it is consistent with the model used to characterize behavior and also neural effects of association strength and updating of stimulus-outcome associations.

COPE	Region	Voxels	p-value	z-stat (max)	X (max)	Y (max)	Z (max)

RS Block GLM: Difference between LC and HC items, modulated by difference in transition probability estimates: (LC – HC)*(r_{HC} – r_{LC})	Posterior Cingulate Cortex	1046	9.18E-06	3.68	14	-56	20
	Middle temporal gyrus/ Hippocampus/ Amygdala/ Perirhinal cortex	739	0.00027	4.04	58	-6	-22
	Temporal parietal junction area	595	0.00154	4.4	-42	-54	22
	Inferior temporal gyrus	490	0.00603	3.72	44	-56	-10
	Hippocampus/ Para-hippocampal gyrus	433	0.0131	3.48	-34	-14	-20
Choice Feedback* Identity update (signed D_{kl})	PCC	3675	3.71E-16	4.37	-4	-52	6
	Lateral occipital cortex (LOC, superior)	1689	4.61E-09	4.3	-30	-86	28
	Insula/inferior temporal gyrus (ITG, posterior)	544	0.00159	4.13	-38	-24	2
	lOFC/vlPFC	499	0.00298	3.85	-36	30	-16
	ACC (cingulate gyrus)	457	0.00547	4.04	0	20	26
Choice Feedback * Identity update (unsigned D_{kl})	Dorsolateral frontal cortex	904	1.84E-05	4.73	52	10	34
	Intraparietal Area	332	0.0388	3.94	42	-44	44
Choice Feedback * Reward payout	Intraparietal Area	1414	1.01E-09	4.45	-46	-50	46
	Supramarginal gyrus	1076	5.96E-08	4.31	40	-46	26
	Dorsolateral frontal cortex (superior frontal gyrus)	1046	1.19E-07	4.01	-20	28	50
	Sensorimotor cortex (postcentral gyrus)	518	0.000306	3.59	-66	-10	16
	Lateral occipital cortex (LOC, superior)	399	0.00253	3.84	-36	-68	28
	Sensorimotor cortex (postcentral gyrus)	321	0.0113	3.76	50	-20	48
	Intraparietal Area	294	0.0195	3.54	46	-42	58

	Hippocampus	261	0.0385	4.28	-32	-26	-12
	Insular cortex (posterior)/ Putamen	255	0.0437	3.78	-30	-22	10

Table S1, related to Figure 3 and Figure 4. Full report of contrasts of interest. Details of activations for each contrast reported in the Results. All reported activations survive a cluster-forming threshold across the whole brain of Z>2.3, and a family-wise error rate of *p*=0.05. Coordinates refer to standardized Montreal Neurological Institute (MNI) 152 space.

	**CSS Peaks in Medial Temporal Lobe**			
		**Hippocampus**	**Amygdala**	**Perirhinal Cortex**
**Choice Feedback* Identity update (signed D_{kl})**	**lOFC**	*t(21)=2.5, p=0.01	*t(21)=1.85, p=0.04	t(21)=1.51, p=0.07
	**ACC**	t(21)=1.26, p=0.11	t(21)=-0.55, p=0.29	t(21)=0.81, p=0.21
	**PCC**	*t(21)=2.04, p=0.03	t(21)=0.27, p=0.39	t(21)=0.56, p=0.29
	**ITG**	t(21)=0.78, p=0.22	t(21)=-0.20, p=0.42	t(21)=1.18, p=0.13
	**LOC**	t(21)=-0.46, p=0.33	t(21)=-1.25, p=0.11	t(21)=0.90, p=0.19

Table S2, related to Figure 5. For completeness, we performed *post-hoc* tests using ROIs from each region showing signed identity update effects at choice feedback (rows) as the predictor and the single-trial change to CSS in each peak within the medial temporal lobe as the dependent variable (columns) within separate general linear models. Choice feedback-locked responses from each CSS ROI, model-derived stimulus-outcome updates, and reward payouts were included in the general linear model as covariates of no interest. T-statistics result from one-sample t-tests; * denotes effects at p<0.05 uncorrected for multiple comparisons.

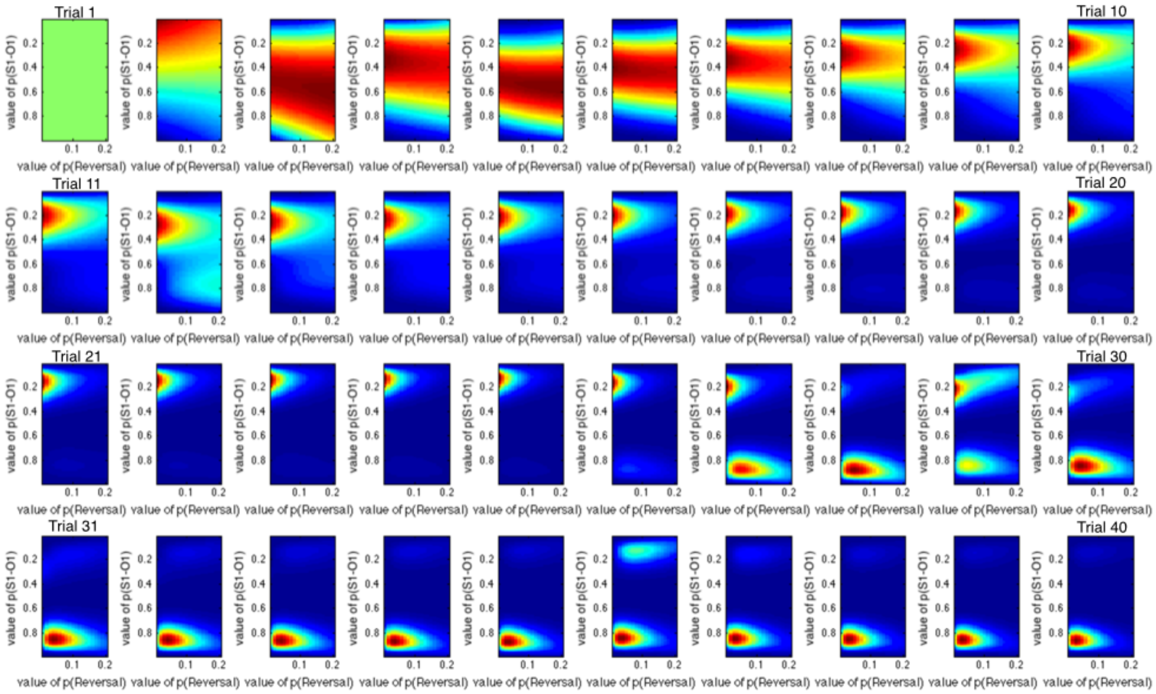

Figure S1, related to Figure 2. Reversal model joint distributions. Heatmaps depict the probability mass of each value of the joint probability distribution over reversal probabilities (abscissa) and stimulus-outcome transition probabilities (ordinate) for the first 40 choice trials. To produce the plots in figure 2, we marginalized over reversal probabilities.

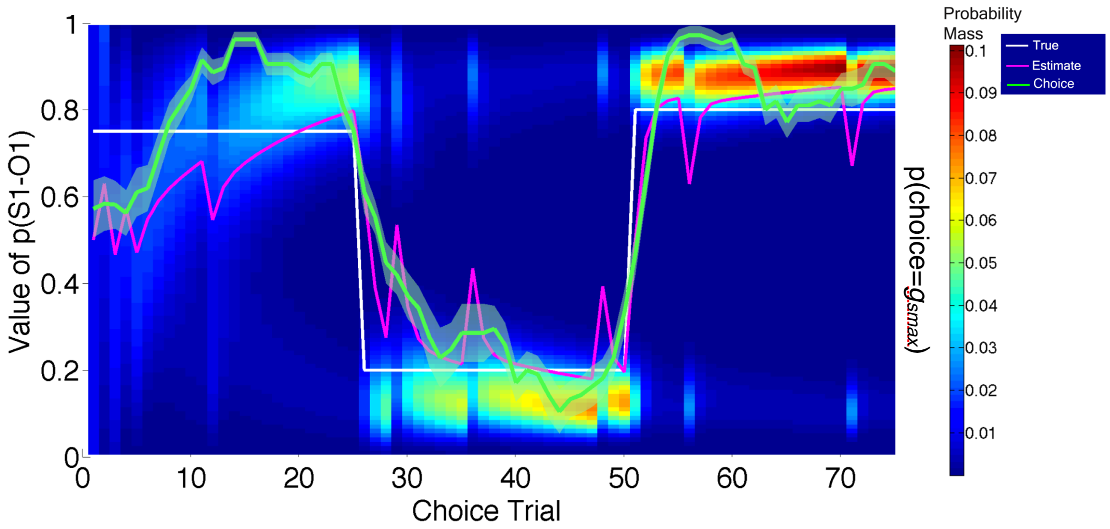

Figure S2, related to Figure 2. Relationship between transition probabilities and stimulus choices. To illustrate the relationship between transition probability estimates and subject choices, the mean probability (bright green) +/- group SEM (light green shadow) of selecting the stimulus with the maximal subjective expected value (g_{smax}) is overlaid onto the model estimates also shown in Figure 2A. Choice probability is computed from a running average with a centered five-choice window. For the middle 25 choice trials (trials 26-50), 1 – p(choice=g_{smax}) is plotted to facilitate comparison with

the mean S1-O1 transition probability estimate (magenta). The true data generating S1-O1 transition probability is shown in white. Potential reward payouts, which are combined with transition probabilities to determine subject choices, are not shown on this plot. These mainly account for the difference between green and magenta curves. Conventions are otherwise the same as Figure 2A.

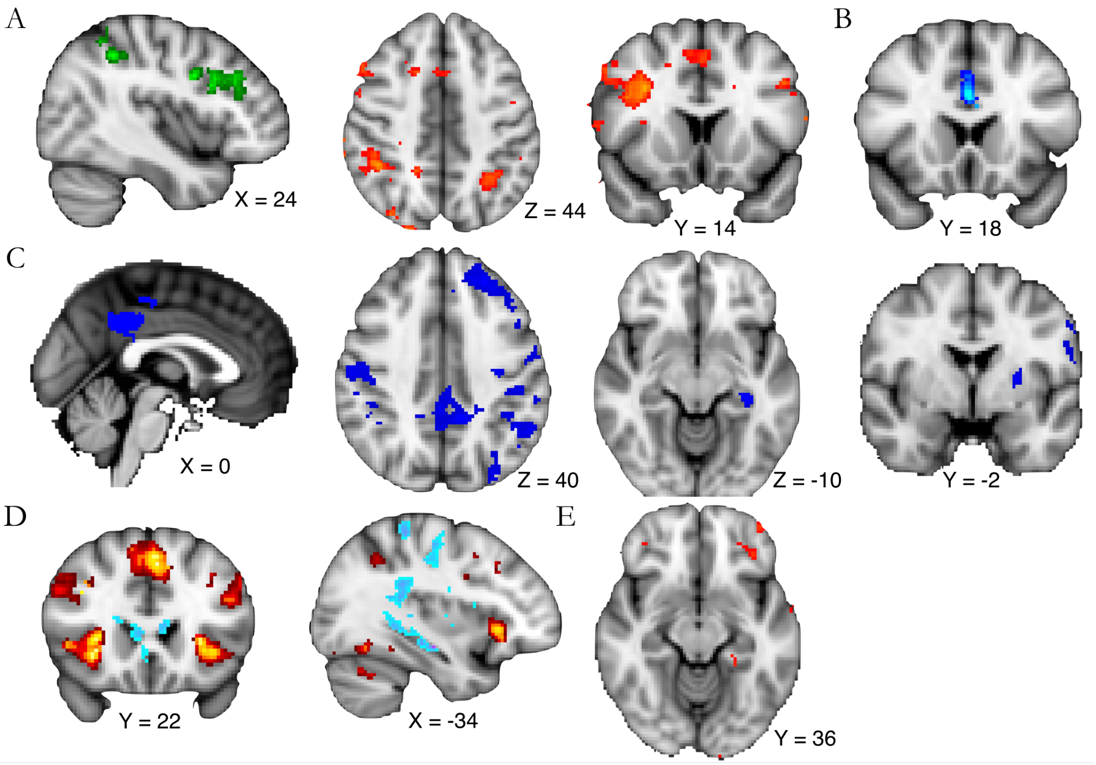

Figure S3, related to Figure 4. Whole-brain effects of different contrasts at choice feedback. (A) Left: Significant effects (cluster corrected) in a frontoparietal network related to the effect of DKL (unsigned) at choice feedback. Middle, right: Activations in contralateral IPS and DLPFC and pre-SMA are displayed at a reduced threshold of Z>2.33 p<0.01 uncorrected for display purposes. (B) Significant effect (cluster corrected) in ACC at choice feedback relating to the effect of D_{KL} (signed), localized ventrally to the pre-SMA region shown in (A). (C) Significant effects (cluster corrected) relating to signed reward payout at choice feedback in posterior cingulate cortex, hippocampus, putamen, and sensorimotor cortex. (D) Z-statistic map relating to the contrast of main effects between non-preferred and preferred outcomes (O_{np} - O_{p}) at feedback. Hot and cool colors denote positive and negative effects, respectively, thresholded at Z>3.1, p<0.001 uncorrected for display purposes. Hot colors indicate greater activity for non-preferred (and hence less expected) than preferred (and hence more expected) outcomes, while cool colors indicate the reverse. (E) Z-statistic map relating to the effect of LC – HC main effects (i.e. categorical difference between low and high contingent transitions, not modulated by trial-by-trial association strength), showing differential activation in left lOFC and hippocampus/parahippocampal gyrus, thresholded at Z>2.33, p<0.01 uncorrected for multiple comparisons.

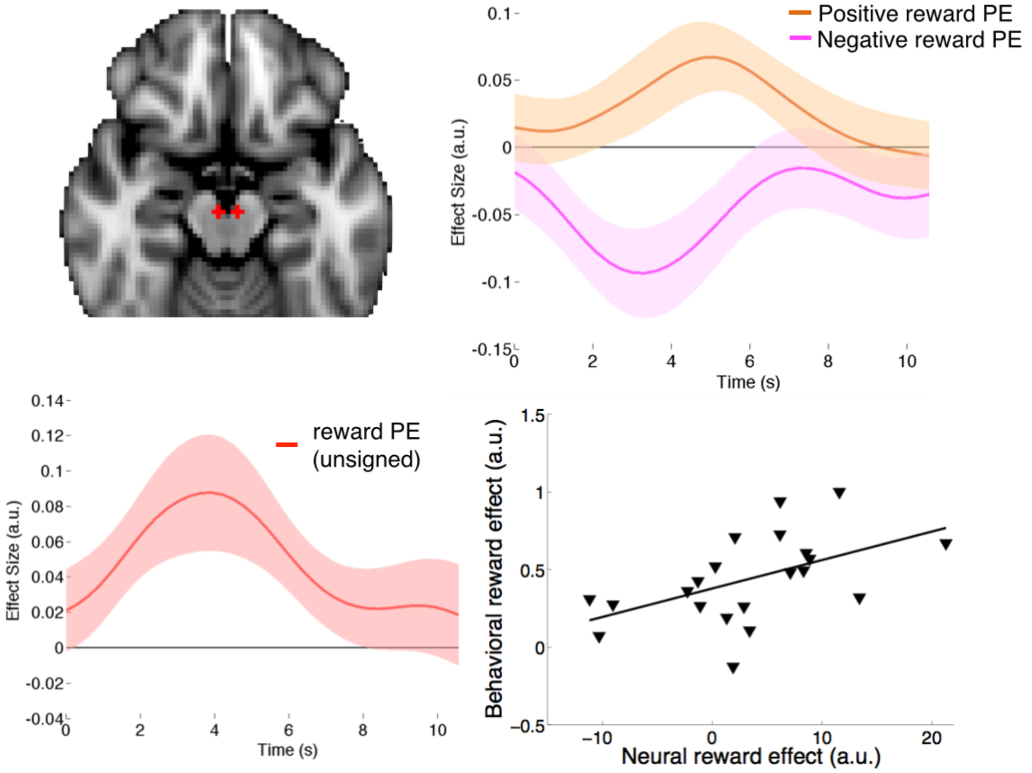

Figure S4, related to Figure 4. VTA effects of reward prediction errors. Upper left: ROIs in VTA. Bottom left: Timecourse of unsigned reward prediction error effect in left VTA ROI. Upper right: Timecourse of positive (orange) and negative (magenta) reward prediction error effects, defined with respect to preferred and unpreferred options. Bottom right: Scatterplot depicts relationship between behavioral effect of reward payout depicted in Fig 2B and neural effect of unsigned reward prediction error in left VTA. Conventions are the same as in Figure 2 in the main text.

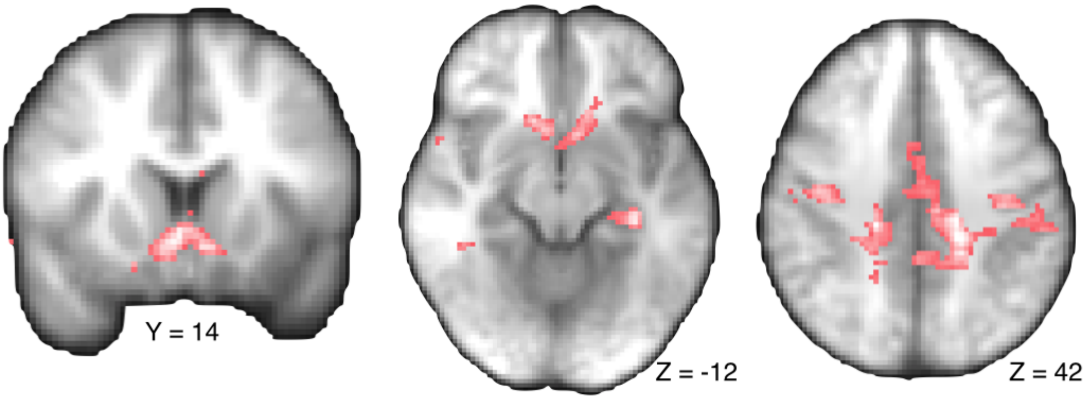

Figure S5, related to Figure 4. Whole-brain effects of reward prediction errors. Whole brain cluster-corrected Z-statistic map relating to the effect of reward prediction error at choice feedback. Activations shown survive a cluster-forming threshold across the whole brain of Z>2.3, and a family-wise error rate of *p*=0.05.

Supplemental References

1 Behrens, T. E., Woolrich, M. W., Walton, M. E. & Rushworth, M. F. Learning the value of information in an uncertain world. *Nat Neurosci* **10**, 1214-1221 (2007).
